# Use of zinc oxide nanoparticles in the growing rabbit diets to mitigate hot environmental conditions for sustainable production and improved meat quality

**DOI:** 10.1186/s12917-022-03451-w

**Published:** 2022-09-21

**Authors:** Ahmed A. A. Abdel-Wareth, Shimaa A. Amer, Muhammad Mobashar, Hazem G. M. El-Sayed

**Affiliations:** 1 Department of Animal and Poultry Production, Faculty of Agriculture, South Valley University, Qena, 83523 Egypt; 2 Department of Nutrition and Clinical Nutrition, Faculty of Veterinary Medicine, Zagazig University, Zagazig, 44511 Egypt; 3 Department of Animal Nutrition, The University of Agriculture, Peshawar, Pakistan; 4 Regional Centre for Food and Feed (RCFF), Agricultural Research Centre, Cairo, 12619 Egypt

**Keywords:** Growth performance, Meat Quality, Rabbits, Serum metabolic profile, Zinc Nanoparticles

## Abstract

**Background:**

This study evaluated the modulatory effects of zinc oxide nanoparticles (ZnO-NPs) supplementations on the productive performance, blood biochemistry, carcass criteria, and meat quality of White New Zealand rabbits reared under hot conditions. A total of 125 White New Zealand male rabbits (body weight, “650 ± 11”, 30 days old) were assigned to five treatment diets: basal diets supplemented with ZnO-NPs at 0, 20, 40, 60, or 80 mg/kg for 60 days. Each treatment was replicated 25 times with one rabbit each.

**Results:**

The body weight (BW), BW gain, and feed intake linearly increased with zinc oxide nanoparticle supplements. Supplementation of ZnO-NPs at 20, 40, 60, and 80 mg/kg significantly improved (linear, *P* < 0.05) the feed conversion ratio compared to the control group. Moreover, supplementation of ZnO-NPs at these inclusions 20, 40, 60, and 80 mg/kg significantly (*P* < 0.05) decreased the serum cholesterol, alanine aminotransferase, and aspartate aminotransferase, creatinine, and urea compared to control group. The lipid oxidation was lower, and the water holding capacity of rabbit meat was improved (*P* < 0.001) in rabbits fed on 20, 40, 60, and 80 mg/kg ZnO-NPs supplemented diets compared to control.

**Conclusion:**

The results suggested that dietary supplementation of ZnO-NPs (20–80 mg/kg) can mitigate the negative impacts of heat stress on rabbit performance and health. Its supplementation improved growth performance and meat physicochemical properties, and blood biochemistry parameters of White New Zealand rabbits.

## Background

The ideal ambient temperature for efficient rabbit production is between 15 and 20 °C, and the ideal relative humidity (RH%) for rearing rabbits after weaning is between 60 and 70% [1]. Since they are isothermal animals and cannot produce thermoregulatory sweat [2], which reduces their capacity to expel excess body heat, rabbits are more susceptible to high ambient temperature conditions. Therefore, an increased respiratory rate (RR) becomes one of the mechanisms required to cause evaporative heat loss when behavioural changes do not affect the maintenance of home heat [3]. Due to lipid peroxidation brought on by DNA, protein, and cell phospholipid membrane damage, heat stress may increase the formation of reactive oxygen species (ROS) [4–7]. Rabbits are becoming more and more important in the global meat industry, but hot weather conditions have an impact on an animal’s productive physiology and the quality of its meat, and animal mortality rises [8–11]. Since growing rabbits are particularly sensitive to high temperatures and suffer from heat anxiety during the summer, intensive rabbit production is impacted by environmental and nutritional factors [12, 13].

Moreover, Exposure to hot climate conditions has a negative impact on the productive performance of male rabbits [14–16]. In order to lessen the negative effects of heat stress on animals, a variety of strategies are available. In this regard, the use of trace minerals like zinc lessens the harmful effects of heat stress on rabbits by assisting with a variety of physiological and metabolic processes [17].

Zinc (Zn) is one of the essential minerals that can be added as a supplement to animal diets and could improve growth, bone development, enzyme structure, organ functions, and immune system [18–21] and plays a significant role in the metabolism of protein, fat, and carbohydrates [22] and gut health and therefore resulting in better feed efficiency [23]. Most studies have shown slight positive outcomes; however, significant results were rare. Dietary supplementation of 100 mg ZnO could improve rabbits’ production, meat quality, and antioxidant capacity under heat stress [24]. However, the commonly used feed ingredients in rabbit diets have high phytate content that may lower Zn absorption [25].

Recently, nanotechnology and its related products have rapidly progressed in different scientific areas [26]. Nano-minerals interact more efficiently with organic and inorganic materials in the animal body owing to their larger surface area, solid adsorption capacity, and high catalytic efficiency [27, 28]. Nano-minerals can cross the small intestine and circulate into the blood and the internal organs [29]. Therefore, these nanoscale minerals are supposed to be efficient in small quantities, provide better bioavailability, and constantly interact with other elements when fed as an alternative to conventional sources [30].

Zn oxide nanoparticles (ZnO-NPs) (< 100 nm) have been produced as feed additives with unique features and activities, such as increasing the surface area of particles and efficient uptake by cells; therefore, they can improve feed bioavailability compared to other ordinary minerals with larger particle size [30, 31]. Recently, there has been an increase in interest in employing dietary ZnO-NPs to enhance productive performance due to its antioxidant properties, bioavailability, and modulation of rabbit immune [32]. The Zn contents in the rabbit diets ranged from 30 to 110 mg/kg in non-supplemented diets to about 250 mg in a 100 mg ZnO or 60 mg ZnO-NPs/kg supplemented diet [24, 30, 33, 34]. Since there are almost unlimited possibilities concerning the dosage of Zn, more research is warranted to determine the optimal levels and new sources of Zn in rabbit diets under hot climatic conditions. Nanoparticles can be used at lower doses and provide better results than conventional Zn sources. We hypothesized that ZnO-NPs could mitigate the negative impacts of hot environmental conditions on growing rabbits’ productive performance and health. Therefore, this study aimed to investigate the modulatory effects of dietary inclusion of ZnO-NPs on the growth, serum biochemistry, carcass criteria, and meat quality of White New Zealand rabbits reared under hot conditions.

## Results

### Growth performance

The effects of dietary supplementation with 0, 20, 40, 60, or 80 mg/kg levels of ZnO-NPs on growth performance are presented in Table 1. The results revealed that BW was linearly increased with the increasing levels of supplementations of ZnO-NPs at 60 and 90 days of age. Furthermore, supplementations of ZnO-NPs linearly increased DBW gain during 30–60, 60–90, and 30–90 days of age. However, daily feed intake did not significantly affect by supplementations. Interestingly, supplementation of ZnO-NPs at 20, 40, 60, and 80 mg/kg improved considerably (linear, *P* < 0.05) the feed conversion ratio compared to the control group under hot environmental conditions during 30–60, 60–90, and 30–90 days of ages.

**Table 1:** Effects of ZnO-NPs on growth performance of male rabbits

Items	ZnO-NPs levels mg/kg					SEM	*P*-value	
	0	20	40	60	80		Lin	Quad
Bodyweight, g								
30 days	628	625	631	624	623	11	0.158	0.333
60 days	1225^b^	1281^a^	1289^a^	1308^a^	1292^a^	23	0.005	0.042
90 days	2030^b^	2119^a^	2145^a^	2186^a^	2158^a^	22	0.010	0.033
Daily weight gain, g								
30–60 days	19.90^b^	21.87^a^	21.93^a^	22.80^a^	22.30^a^	1.06	0.008	0.012
60–90 days	26.83^b^	27.93^a^	28.53^a^	29.27a	28.87^a^	1.01	0.001	0.015
30–90 days	23.37^b^	24.90^a^	25.23^a^	26.03^a^	25.58^a^	0.91	0.003	0.018
Daily feed intake, g								
30–60 days	81.37	83.94	84.63	84.67	83.67	3.51	0.612	0.66
60–90 days	97.92	96.40	97.10	98.70	97.70	3.83	0.174	0.547
30–90 days	89.65	90.17	90.87	91.69	90.69	2.72	0.124	0.244
Daily feed conversion ratio								
30–60 days	4.089^b^	3.839^a^	3.859^a^	3.714^a^	3.752^a^	0.08	0.004	0.022
60–90 days	3.649^b^	3.451^a^	3.403^a^	3.372^a^	3.385^a^	0.04	0.002	0.017
30–90 days	3.836^b^	3.621^a^	3.601^a^	3.522^a^	3.545^a^	0.03	0.001	0.019

^a-c^ Means (*n* = 25) not sharing a common superscript in a row are significantly different (*P* < 0.05). *SEM* standard error of means, *Lin and Quad* linear and quadratic responses, respectively, to supplementation levels ZnO-NPs

### Serum metabolic profile

Effects of 0, 20, 40, 60, and 80 mg/kg of ZnO-NPs on the kidney and liver function markers and cholesterol in serum of the tested rabbits under hot conditions were listed in Table 2. Supplementations of ZnO-NPs to rabbit diets significantly exhibited lower serum levels of GOT and GPT activities than the control. Moreover, serum creatinine and urea were linearly decreased (*P* < 0.05) in response to ZnO-NPs supplementations in the comparison group. Serum cholesterol was significantly decreased (linear *P* < 0.01) with the increasing levels of ZnO-NPs treatments compared to the control. The lowest cholesterol levels, urea, creatinine, GOT, and GPT (quadratic, *P* < 0.01) were obtained with the supplementation level of 60 mg/kg ZnO-NPs compared to other treatments.

**Table 2:** Effects of ZnO-NPs on serum metabolic profile of growing rabbits

Items	ZnO-NPs levels mg/kg					SEM	*P*-value	
	0	20	40	60	80		Lin.	Quad.
Blood,								
GOT, U/L	27.65^a^	23.61^b^	23.26^b^	22.05^b^	23.03^b^	0.218	0.001	0.008
GPT, U/L	45.32^a^	39.07^b^	38.60^b^	36.08^b^	37.21^b^	1.601	0.008	0.018
Urea, mg/L	45.29^a^	42.08^b^	41.65^b^	38.40^b^	40.19^b^	1.101	0.006	0.012
Creatinine, mg/L	6.160^a^	4.165^b^	3.916^b^	3.412^b^	4.387^b^	0.532	0.002	0.014
Cholesterol, mg/L	85.93^a^	81.57^b^	81.25^b^	79.50^b^	80.27^b^	1.018	0.001	0.021

^a-c^ Means (*n* = 25) not sharing a common superscript in a row are significantly different (*P* < 0.05). *SEM* standard error of means, *Lin and Quad* linear and quadratic responses, respectively, to supplementation levels of ZnO-NPs

### Carcass criteria

Effects of diets supplemented with ZnO-NPs at 0, 20, 40, 60, and 80 mg/kg on carcass criteria of rabbits are presented in Table 3. Compared to the control group, diets supplemented with ZnO-NPs levels increased the dressing %, but the fat% decreased (*P* < 0.01) under hot conditions. The testes% significantly increased with the increasing level of ZnO-NPs up to 60 mg/kg compared to the control group and other treatments. There were no differences in internal organs, including the head, liver, hearts, spleens, kidneys, and small intestine with ZnO-NPs treatments compared to control.

**Table 3:** Effects of ZnO-NPs on carcass criteria of growing rabbits

Items (%)	ZnO-NPs levels, mg/kg					SEM	*P*-value	
	0	20	40	60	80		Lin.	Quad.
Carcass%	60.60^b^	63.40^a^	64.02^a^	65.03^a^	64.07^a^	2.200	0.001	0.041
Fore%	38.42^a^	35.83^b^	34.66^b^	33.85^b^	36.32^b^	1.133	0.001	0.006
Loin%	22.01	23.42	22.51	22.86	22.33	2.302	0.055	0.056
Hind%	40.13^b^	41.25^ab^	42.64^a^	43.84^a^	42.73^a^	1.015	0.002	0.041
Fat%	3.882^a^	2.725^b^	1.980^c^	1.822^c^	1.881^c^	0.621	0.001	0.001
Liver%	3.671	3.472	3.462	3.592	3.413	0.821	0.067	0.131
Heart%	0.772	0.782	0.767	0.788	0.774	0.542	0.412	0.808
Spleen%	0.142	0.139	0.153	0.143	0.138	0.134	0.797	0.679
Kidneys%	0.138	0.133	0.135	0.134	0.133	0.292	0.712	0.878
Head%	6.622	6.578	6.631	6.463	6.773	0.121	0.810	0.081
Testes%	1.412^b^	1.689^a^	1.752^a^	1.947^a^	0.767	0.127	0.008	0.024

^a-c^ Means (*n* = 25) not sharing a common superscript in a row are significantly different (*P* < 0.05).(*n* = 25). *SEM* standard error of means, *Lin and Quad* linear and quadratic responses, respectively, to supplementation levels of ZnO-NPs

### Meat quality

The effects of ZnO-NPs on meat quality properties of growing rabbits are shown in Table 4. Supplementation of ZnO-NPs at 0, 20, 40, 60, and 80 mg/kg significantly improved (linear, *P* < 0.05) WHC with increasing levels of supplementations. The dietary supplementation of ZnO-NPs in the rabbit’s diet also significantly improved TBARS values (linear, *P* < 0.01) compared to control. However, ZnO-NPs supplementations did not affect the protein, fat, moisture, and Zn contents compared to the control group in the foreleg and hind carcass.

**Table 4:** Effects of ZnO-NPs on meat quality parameters of growing rabbits

Items	ZnO-NPs levels, mg/kg					SEM	*P*-Value	
	0	20	40	60	80		Lin	Quad
Hind leg								
WHC %	25.55^b^	27.06^a^	27.67^a^	27.74^a^	27.68^a^	1.945	0.008	0.081
pH	5.734	5.741	5.753	5.723	5.726	0.582	0.143	0.916
TBARS	1.514^a^	1.332^b^	1.288^b^	1.285^b^	1.313^b^	0.205	0.002	0.058
Protein %	21.56	21.85	21.99	22.12	21.97	0.264	0.077	0.631
Fat%	2.388	2.444	2.439	2.524	2.608	0.359	0.052	0.678
Moisture%	74.24	74.69	74.66	74.57	74.51	0.249	0.141	0.791
Zn, mg/kg	10.24	10.83	11.13	11.57	11.56	1.768	0.087	0.675
Loin								
WHC %	24.55^b^	26.76^a^	27.16^a^	28.24^a^	27.25^a^	0.687	0.021	0.032
pH	5.734	5.741	5.723	5.744	5.754	0.441	0.067	0.089
TBARS	1.624^a^	1.412^b^	1.410^b^	1.395^b^	1.413^b^	0.045	0.006	0.079
Protein %	21.33	21.88	21.95	22.17	21.87	0.264	0.096	0.431
Fat%	5.578	5.674	5.635	5.575	5.618	0.247	0.674	0.897
Moisture%	72.44	72.55	72.43	72.45	72.51	0.376	0.434	0.989
Zn, mg/kg	9.567	10.34	11.06	11.32	11.45	1.345	0.098	0.567

^a-c^ Means (*n* = 25) not sharing a common superscript in a row are significantly different (*P* < 0.05). *SEM* standard error of means, *Lin and Quad* linear and quadratic responses, respectively, to supplementation levels ZnO-NPs, *WHC* water holding capacity, *TBARS* the thiobarbituric acid-reactive substances

## Discussion

Rabbits are sensitive to heat stress and difficult to reduce their body heat because they have few sweat glands [12]. Hot climatic conditions affect animals’ productivity, meat quality, and physiological states [8]. Zn is essential and has important biological functions for productive animal performance and meat quality under hot conditions [16, 19]. NRC [33] has recommended that about 50 mg/kg Zn is required in growing rabbits’ basal diet. The current study evaluated the modulatory effects of zinc oxide nanoparticles (ZnO-NPs) supplementations on the productive performance, blood biochemistry, carcass criteria, and meat quality of White New Zealand rabbits reared under hot conditions. The results showed that dietary ZnO-NPs up to 80 mg/kg improved growth performance and FCR of growing rabbits reared under hot conditions. The obtained results suggested increased bioavailability and improved absorption of nano-zinc, which are similar to [35, 36]. Compared with inorganic zinc sources, ZnO-NP can modify mineral accumulation due to its high bioavailability [37]. Dietary zinc is vital for good biological functions of the animal, such as growth [21, 38], DNA synthesis, and cell division [39], enhancing the animal’s immunity [36]. Zn plays a significant role in the metabolism of carbohydrate and protein synthesis as well as lipid [18] through its essential part in enzyme actions as a component of several Zn metalloenzymes which are involved in many biological functions such as digestion and absorption and maintain the structural integrity of proteins [18, 40]. ZnO could alleviate heat stress by improving antioxidant properties and reducing heat shock responses [41]. These results are identical to Pathak et al. [42] reported that birds supplemented with a nano-zinc/kg diet had increased weight gain and better FCR than the control group. Also, El-Katcha et al. [43] found that broiler chickens’ final body weight, BWG and FCR, was improved by supplementation of nano-zinc (30, 45, and 60 mg/kg) compared with the control group. Hassan et al. [24] reported that adding 100 mg/kg of zinc oxide to the diet increased the performance of growing rabbits under heat stress.

In the current study, dietary supplementation of ZnO-NPs source improved productive performance of growing rabbits reared under hot conditions. In comparison with other sources, Hassan et al. [30] have noted that feed consumption was significantly decreased in rabbit-fed diet supplemented with organic Zn at 75 and 100 mg/kg compared to the control group. Selim et al. [19] have found that the growth performance of rabbits fed a diet including ZnO at 50, 100, 200, and 400 mg/kg was non-significantly different. On the other hand, Hassan et al. [30] observed a significant improvement in the growth performance and feed intake when rabbits consumed different levels of ZnO-NPs at 30 and 60 mg/kg compared to the control group. The variation in the results could be related to supplementation levels, sources, method, strains, rabbit age, and the duration of the experiments.

In the current study, supplementation of ZnO-NPs in rabbit diets significantly decreased serum cholesterol levels, GOT, GPT, and creatinine activities compared to control. Interestingly, the lowest creatinine, GOT, and GPT levels were obtained at supplementation level of ZnO-NPs at 60 mg/kg compared to control and other treatments. *Reducing* the level of these enzymes indicates better *liver function* [44]*.* These improvements in serum biochemistry of male rabbits may be attributed to the fact that Zn is a component of Zn metalloenzymes and maintain the structural integrity of proteins [18]. Heat stress promotes ROS production [6] that is associated with apoptosis and various diseases [5] due to damage to DNA, proteins, and cell phospholipid membranes caused by lipid peroxidation [4]. ZnO-NPs could mitigate the adverse effects of heat stress on the animal’s health through their crucial roles in cell protection from ROS by lowering free radicals and inhibiting lipids peroxidation [45]. The lower total cholesterol in the ZnO-NPs supplemented groups may be that zinc reduces lipolysis in adipose tissue, leading to the breakdown of triglycerides stored in fat cells and release of fatty acids and glycerol [46]. Al-Sagheer et al. [47] reported no significant effect of ZnO (100 mg/kg) on the serum level of GOT in rabbits.

The present study showed that ZnO-NPs supplemented with the rabbit diets at 60 mg/kg improved the dressing-% and testes%. In contrast, the fat% decreased without significant changes in internal organs compared to the control group. These improvements may be due to the possible nutritional and physiological functions of ZnO-NPs as feed additives for growing rabbits under heat stress conditions [24]. Moreover, dietary zinc is vital for good biological functions of the animal, such as growth [21, 38], DNA synthesis, and cell division [39]. The reduced fat content in the meat may be due to Zn being a cofactor in the biological function as a divalent cation primarily when bound to enzymes, other proteins and plays an essential protective role in the regulation of lipid accumulation and metabolism [40]. Amer et al. [48] showed increased hot carcass weight, dressing, and total edible parts% in rabbits fed Zn supplemented diets at 50, 75, and 100 mg/kg compared with the control group. In contrast, Selim et al. [34] reported no change in carcass traits in rabbits fed diets supplemented with a 50, 100, 200, or 400 mg zinc oxide /kg diet.

Supplementation of ZnO-NPs at 0, 20, 40, 60, and 80 mg/kg significantly improved WHC and TBARS of rabbits with increasing levels of supplementations compared to control. Limited information exists regarding the effects of ZnO-NPs or ZnO sources on the meat quality of rabbits. The results can be attributed to the fact that zinc has an antioxidant activity by inhibiting the oxidation of macromolecules such as proteins and DNA, in addition to the inhibition of the inflammatory response, which ultimately leads to the downregulation of ROS production [49]. Furthermore, a better WHC may be caused by decreased protein de-naturalization by microbes or other environmental factor [50, 51]. The addition of Zn in rabbits’ diet helps deposit it in the meat and thus contributes to human requirements of Zn. In addition, improving WHC can increase the shelf life of meat and customer acceptance [50, 51]. In the current study, the pH and proximal compositions of protein, fat, moisture, and Zn in the hind leg and lion muscles were similar in all treatments. They were maintained regardless of the inclusion of Zn [52, 53]. Luis-Chincoya et al. [52] reported that rabbits’ meat quality, including pH, WHC, protein, fat, and moisture, showed no differences between the Zn supplementation and control treatment. Chrastinová et al. [18] showed that the pH of rabbit meat is not influenced by different levels of Zn. Supplementations of Zn oxide to rabbit diets did not show any differences in control [53]. These results could suggest that the ZnO-NPs source is more available in the small intestine than organic and inorganic sources.

## Conclusion

Dietary supplementation of ZnO-NPs (20–80 mg/kg) could mitigate the negative impacts of heat stress on rabbit performance and health. Its supplementation could improve growth performance, blood biochemical, carcass criteria, fat, and meat quality in male rabbits reared under hot climatic conditions. However, the limitation of this study is that it did not assess the gene expression of antioxidant enzymes and the genes involved in lipid, carbohydrates, and protein metabolism. Therefore, it is recommended to consider the analysis of these genes in the future.

## Methods

### Animal ethics

This study was conducted in a poultry research unit at the Department of Animal and Poultry Production, South Valley University, Egypt, to investigate the modulatory effects of dietary inclusion of ZnO-NPs on the growth, serum biochemistry, carcass criteria, and meat quality of White New Zealand rabbits reared under hot conditions. All methods and procedures used in this study were approved by the Authors’ institution (Department of Animal and Poultry Production, South Valley University, Egypt) Ethics Committee (Approval No. SVUAGR 6/2021). All methods in the study were carried out in accordance with relevant institutional guidelines and all animal experiments were performed following the ARRIVE guidelines.

### ZnO-NPs preparation

**Fig. 1:**
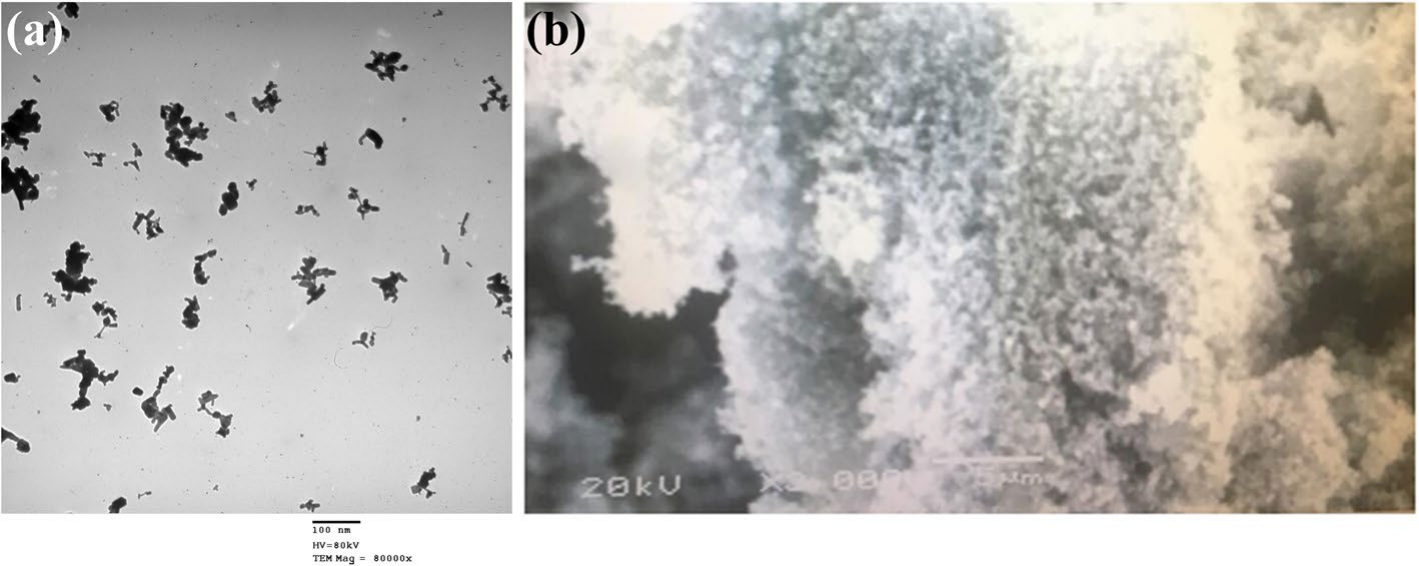
Transmission electron micrographs (TEM) (**a**) and scanning (SEM) (**b**) of synthesized zinc oxide nanoparticles (ZnO-NPs); 1–100 nm, HV = 80 kv, and TEM Mag = 8000 x

The structural morphology of the particle of ZnO-NPs (Cas no.1314-13-2, Sigma-Aldrich, Steinheim, Germany) was determined using scanning and transmission electron microscope (SEM and TEM) (Fig. 1a, b).

### Experimental animals, management, and design

One hundred twenty-five male white New Zealand rabbits (body weight, 650 ± 60 g; 30 days old) were procured from a commercial rabbit producer (South Valley, Egypt) for the experiments. The rabbits were kept at an average temperature of 39 ± 2 °C, a 45–50% relative humidity, and temperature humidity index (THI) 34–36 (Table 5) with a 16 h light and 8 h dark regime in an open house system (naturally ventilated room by windows and ceiling fans) during the experimental period. In-house temperature and relative humidity were recorded three times a day. Rabbits were kept for a 7-day adaptation period before the start of the experiment. Fresh water was available for the rabbits. The animals were raised in individual cages under exemplary management, hygiene, and environmental conditions throughout the experiment. All rabbits remaining after the end of the study were released.

**Table 5:** Temperature, humidity, and temperature humidity index (THI)

Items	Period (days)					
	10	20	30	40	50	60
T (db °C)	40.10	39.20	39.10	38.50	38.20	39.10
RH%	48.00	49.00	46.00	48.00	50.00	45.00
THI	35.96	35.28	34.97	34.62	34.51	34.89

*T, db°C* dry bulb temperature in degrees Celsius, *RH* relative humidity percentage, *THI* relative temperature humidity index

Rabbits were assigned to five treatment diets with ZnO-NPs inclusion at 0, 20, 40, 60, or 80 mg/kg for 60 days. The analyzed zinc contents ZnO-NPs treatment diets were 89, 111, 142, 167, and 188 mg/kg, respectively. Each treatment has 25 replicates with one rabbit each. Rabbits were reared under similar conditions and were offered pelleted diets for ad libitum during the experimental period of 60 days. Freshwater was available for ad libitum consumption. The diets were formulated according to [33]. The ingredients and approximate chemical analysis of the basal diet are presented in Table 6.

**Table 6:** Ingredients composition (as-fed basis) of the basal diet

Ingredients	g/ kg	Chemical composition analyzed	(g/ kg, as fed)
Corn	310	Dry matter	936
Wheat bran	200	Gross Energy (MJ/kg DM)	12.3
Soybean meal (440 g/kg CP)	190	Crude protein	178
Wheat straw	120	Ether extract	39.7
Lucerne hay	60	aNDFom	392
Rice bran	40	ADFom	232
Linseed straw	28	ADL	69.5
Sunflower meal	25	Ash	92.82
Limestone	20	Calcium	15.80
Sodium chloride	3	Phosphorus	7.73
Vitamin-mineral premix^*^	3	Zinc	45
Dl-Methionine	1		

^*^ Per kg of diet: vitamin A 10,000 IU, vitamin D3 900 IU, vitamin E 50.0 mg, vitamin K 2.0 mg, vitamin B1 2.0 mg, folic acid 5.0 mg, pantothenic acid 20.0 mg, vitamin B6 2.0 mg, choline 1200 mg, vitamin B12 0.01 mg, niacin 50 mg, biotin 0.2 mg, Cu 0.1 mg, Fe 75.0 mg, Mn 8.5 mg, ZnO 20 mg. *aNDFom* neutral detergent fibre, *ADFom* acid detergent fiber, *ADL* acid detergent lignin

### Growth performance

The initial body weight (BW), final BW, and feed intake of rabbits were measured on 30, 60, and 90 days of age, respectively. Feed conversion ratio (FCR) and BW gain were calculated at 30–60, 60–90, and 30–90 days. Mortality or any signs of diarrhea were recorded daily as it occurred. Additionally, the amount of feed consumed in each cage between weighings was calculated by measuring the feed residue on the day the rabbits were weighed. The weight of daily feed ingested divided by the BW increase per cage was used to compute the FCR. Additionally, throughout the whole study period, mortality was monitored every day.

### Chemical analysis

Diets were analyzed for moisture by oven drying (method number 930.15), ash by incineration (method number 942.05), protein by Kjeldahl (method number 984.13), and EE by Soxhlet fat analysis (method number 954.02), calcium (method number 927.02) and phosphorous (method number 935.59). The concentrations of neutral detergent fiber (NDFom; 6.5.1), acid detergent fiber (ADFom; 6.5.2), and acid detergent lignin (ADL; 6.5.3) were analyzed as described by the AOAC International Method [54]. The gross energy was measured by adiabatic bomb calorimetry. The Zn concentrations were analyzed by using atomic absorption spectrophotometer.

### Serum metabolic profile

At the termination of the trial at 90 days of age, animals were fasted for 12 hours, and blood samples were taken from five rabbits selected randomly from each experimental group after euthanasia by cervical dislocation according to AVM Association [55]. Blood samples were left to coagulate at room temperature or in the refrigerator for 1 hour and then centrifuged at 3000 RPM for 15 minutes. The clear supernatant serum was transferred into dry, sterile, labeled stopper vials and used to perform the clinical-biochemical tests. Colorimetric diagnostic kits of spectrum-bioscience (Egyptian Company for Biotechnology, Cairo, Egypt) were used for measuring the serum total cholesterol (TC) following the methods of Allain et al. [56]. The serum levels of creatinine and uric acid were measured by an automatic biochemical analyzer (Robotnik Prietest ECO-India) [57, 58]. The method of Reitman and Frankel [59] was used to estimate serum levels of aspartate aminotransferase (GOT) and alanine aminotransferase (GPT).

### Carcass criteria

At the termination of the experimental period, five rabbits from each experimental group were randomly selected, fasted all night, weighed, and euthanized by cervical dislocation according to AVM Association [55]. Carcass criteria as dependent variables were hot carcass weight, dressing percentage, fore parts, loin, and hind parts weight, expressed as a percentage of the slaughter weight. The kidney, liver, lungs, heart, spleen, and testes were also expressed as a percentage of the slaughter weight. The relative weight of some organs was calculated according to Abd El-Samee [60].

### Meat quality

The physicochemical properties of meat, including pH values and water holding capacity (WHC) in loin and leg samples, were measured according to Nakamura and Katoh [61]. The WHC of muscle tissue was estimated by centrifuging 1 g of muscle, placed on tissue paper inside a tube, at 1500×g for 4 min. The water remaining after centrifugation was quantified by drying the samples at 70 °C overnight. The WHC was calculated as follows: WHC (%) - 100 × (Weight after centrifugation - Weight after drying)/Initial weight. Also, lipid oxidation was measured by the thiobarbituric acid-reactive substances (TBARS) method described by Lee et al. [62]. Protein, fat, and moisture were determined as described by the AOAC [54]. The determinations of Zn in loin and leg samples were measured according to the methodology of Hassan et al. [30].

### Statistical analysis

The statistical analysis was applied using a completely randomized design (CRD) and the general linear model (GLM) procedure of SAS [63]. Pens were the experimental units for all analyses. To determine the linear and quadratic effects of the increasing inclusion levels, orthogonal polynomial contrasts were used in statistical analysis. To compare means, Duncan’s multiple range test was utilized. Significance was declared at *P* < 0.05.

#### Statistical model

$$\mathrm{Yik}=\mathrm{U}+\mathrm{Ti}+\mathrm{Eijk}$$

Where; Yik = observed value of the response variable, U = observed mean for the response variable, Ti = the fixed effect of the treatment group, Eijk = random error.

#### Calculation of THI and Determination of heat stress index (HSI)

The rabbit temperature relative humidity index (THI) was calculated using Marai et al. [64]. Equations of THI, according to Ogunjimi et al. [65], are

$$THI=t-\left[\left(0.31-0.31\left(\frac{RH}{100}\right)\right)\left(t-14.4\right)\right]$$

Where: t °C = dry bulb temperature in degrees Celsius, and RH = relative humidity percentage/100.From the thermal comfort level of an animal environment according to LPHSI [66], the THI values were classified as follows:

< 27.8 = absence of heat stress; 27.8–28.9 = moderate heat stress; 29.0–30.0 = severe heat stress; > 30.0 = very severe heat stress.

## Data Availability

The datasets generated and/or analyzed during the current study are not publicly available due to institution roles and permission but are available from the corresponding author upon reasonable request.

## Abbreviations

ZnO-NPs: Zinc oxide nanoparticles
SEM and TEM: scanning and transmission electron microscope
BW: body weight
FCR: Feed conversion ratio
aNDFom: neutral detergent fibre
ADFom: acid detergent fiber
ADL: acid detergent lignin
GOT: aspartate transaminase
GPT: alanine transaminase
WHC: water holding capacity
TBARS: the thiobarbituric acid-reactive substances
